# Involved-field radiotherapy in older patients with superficial thoracic esophageal squamous cell carcinoma: long-term outcomes and recurrence patterns

**DOI:** 10.1007/s11604-024-01564-w

**Published:** 2024-04-22

**Authors:** Sawa Kono, Yaichiro Hashimoto, Kenta Ohmatsu, Miki Tsujii, Shigehiko Kuribayashi, Kumiko Karasawa

**Affiliations:** Department of Radiation Oncology, Tokyo Women’s Medical University School of Medicine, 8-1, Kawada-cho, Shinjuku-ku, Tokyo, 162-8666 Japan

**Keywords:** Involved-field radiotherapy, Stage I, Esophageal carcinoma, Older individuals, Chemoradiotherapy

## Abstract

**Purpose:**

An optimal radiotherapy field for superficial esophageal carcinoma is yet to be established. We evaluated the long-term outcomes and recurrence patterns of involved-field radiotherapy (IFRT) in older patients with superficial thoracic esophageal squamous cell carcinoma (ESCC).

**Materials and methods:**

Fifty-four patients (49 men and 5 women; mean age, 77 [range: 66–90] years) who underwent IFRT for superficial thoracic ESCC between January 2003 and January 2019 were retrospectively reviewed. Concurrent chemotherapy was administered at the discretion of the attending physician. The primary endpoint was overall survival. The secondary endpoints were progression-free survival and complete response rate.

**Results:**

The tumors were localized in the upper, middle, and lower thoracic esophagus in 2, 40, and 12 patients, respectively. All patients underwent IFRT using anteroposterior and anterior–posterior oblique opposed beams (off-cord). The prescribed total doses were 50.4, 59.4–61.2, and 66–70 Gy for 6, 40, and 8 patients, respectively. Concurrent chemotherapy was administered to 33 patients. The median follow-up duration was 57 months. The median overall survival was 115 months. The 5-year overall and progression-free survival rates were 71.7% and 60.1%, respectively. Forty-nine patients had a complete response at one month after IFRT (complete response rate: 90.7%). Twenty patients had recurrence; there were 13 in-field and 7 out-of-field recurrence cases. The radiation-related adverse events were generally mild. Grade 3 late toxicity was observed in one patient.

**Conclusions:**

The efficacy of IFRT was suggested to be comparable to that of standard treatments. Therefore, IFRT can be a promising approach for treating superficial ESCC in older adults, especially those with severe comorbidities.

## Introduction

The detection rate of superficial esophageal carcinoma has increased significantly in recent years due to the widespread use of endoscopy and advancements in diagnostic techniques [1, 2]. The treatment approach for superficial esophageal squamous cell carcinoma (ESCC) is based on the depth of tumor invasion and differs between tumors that invade the lamina propria or muscularis mucosa (T1a) and those that invade the submucosa (T1b) [3]. While surgery remains the primary treatment modality for T1b carcinomas, endoscopic intervention is preferred for T1a tumors smaller than 2 cm and those with well-to-moderate differentiation [4]. Endoscopic mucosal resection (EMR) is frequently performed, with or without ablation therapy. As lymph node metastases are rarely observed in patients with lesions located within the epithelium and lamina propria mucosa, EMR is often curative. However, tumors invading the muscular mucosa and those with minor submucosal (up to 200 μm) or lymphovascular invasion carry a risk of lymph node metastasis. Thus, post-EMR surgical intervention is strongly recommended [4].

The incidence of esophageal carcinoma is highest for the older population. According to the Ministry of Internal Affairs and Communications, individuals aged ≥ 65 years accounted for 29.1% of the cases of esophageal carcinoma in Japan in 2022 [5]; this incidence is considered high. Squamous cell carcinoma is the most common histological type of esophageal carcinoma, accounting for 86.7% of cases in Japan, according to the 2015 Japanese Esophageal Association National Registry of Esophageal Cancer [6]. Thoracic esophageal carcinoma, the most common esophageal carcinoma, is found in various esophageal portions [6]. Older patients, particularly those with comorbidities, a history of malignancies, or those who decline surgery, may opt for definitive radiotherapy as an alternative to surgery.

The optimal radiation field has not been established for superficial esophageal carcinomas. Thoracic esophageal carcinoma is characterized by extensive local infiltration and lymph node involvement, attributed to the absence of a serosal envelope and prominent submucosal lymphatic network. Such anatomical arrangement frequently results in concealed micrometastases with recurrence patterns that underrepresent the profound and universally fatal implications of distant relapse [7]. Consequently, standard definitive radiotherapy, covering a broad spectrum of prophylactic lymph node regions, has become the preferred treatment for esophageal carcinomas. Irradiation encompasses three regions from the supraclavicular fossa to those near the celiac artery, and the post-esophagectomy recurrence distribution varies based on the tumor location and histological type. While local recurrence is more common in patients with upper thoracic (Ut) and middle thoracic (Mt) ESCC, distant recurrence is prevalent among those with lower thoracic (Lt) lesions, predominantly adenocarcinomas [8]. However, opinions about its necessity vary, and a consensus is yet to be established. Extensive prophylactic irradiation can lead to severe radiation-induced complications, such as pneumonitis, pleural effusion, and cardiac complications. Furthermore, complications from salvage surgery may occur in patients with an incomplete response or local recurrence after radiation. In recent years, involved-field radiotherapy (IFRT), which targets only the tumor area, has gained attention for treating early-stage cancer. IFRT aims to reduce radiation exposure to the surrounding healthy organs, such as the heart and lungs, in patients with esophageal carcinoma.

This study aimed to comprehensively analyze the long-term outcomes and recurrence patterns after IFRT in older patients with superficial ESCC. Building on our previous work [9, 10], the sample size was increased to enable a more robust evaluation of the observed outcomes and recurrence patterns over time.

## Materials and methods

### Patients

Patients aged ≥ 65 years who were diagnosed with clinical stage I (cT1N0M0) thoracic ESCC and underwent initial treatment with IFRT at our institution between January 2003 and January 2019 were retrospectively reviewed. Staging was based on the 8th edition of the TNM classification [11]. All patients had histologically confirmed squamous cell carcinoma. Each patient underwent chest radiography, chest computed tomography, and esophageal endoscopy with ultrasonography. An endoscopist performed the depth of tumor invasion assessment. Positron emission tomography (PET) was rarely performed for pretreatment diagnosis. We excluded patients after endoscopic treatment as initial treatment. Patients with T1a tumors were not considered eligible for initial endoscopic treatment due to the presence of extensive disease. The study design was approved by our institution's Ethics Review Board (protocol number: 2023-0069), and all participants provided informed consent for this treatment.

### Radiotherapy

A metallic clip was carefully inserted into the proximal and distal areas of the gross tumor or the unstained region, identified using iodine staining, during esophageal endoscopy before radiotherapy. The radiotherapeutic strategies primarily targeted the gross tumor volume (GTV), including the primary tumor volume observed during esophageal endoscopy.

**Fig. 1:**
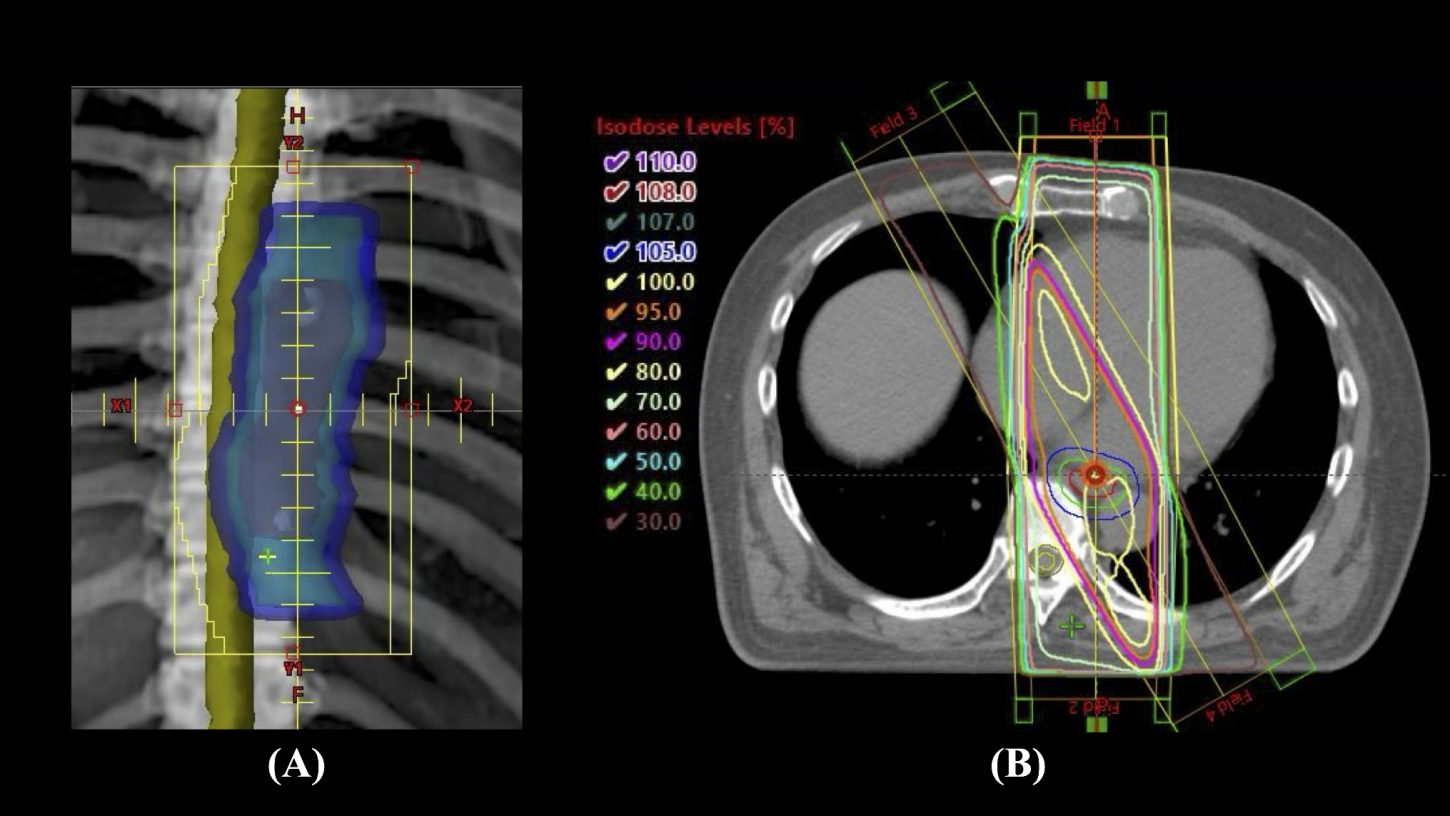
**A** Example of radiation field. *Blue line* planning target volume (PTV) and *green line* clinical target volume (CTV) and *red line* gross tumor volume (GTV). **B** Example of dose distribution

The clinical target volume (CTV) included the metal clip with a 2 cm margin above and below the GTV and a lateral extension of 0.5 cm along the GTV. Adhering to the principles of IFRT, regional lymph nodes were not included within the CTV. The planning target volume (PTV) extended 0.5 cm outward from the CTV in all dimensions. Four radiation portals were used to minimize cardiac exposure, with radiation delivered through the anterior–posterior opposed portals and the anterior–posterior oblique opposed portals to spare the spinal cord. The portals utilized radiation beams of 6–10 Mega Volts. The reference point for the radiation dose was set in the central region of the PTV. We ensured that the maximum dose constraint for the spinal cord did not exceed 46 Gy. All patients underwent IFRT using a three-dimensional conformal radiotherapy technique, avoiding intensity-modulated radiotherapy. Figure 1 shows a typical example of the radiation field and dose distribution.

### Chemotherapy

The attending physicians determined the appropriateness of concurrent chemotherapy. Concurrent chemotherapy was not administered to patients with severe comorbidities or renal dysfunction or those who declined chemotherapy. The primary chemotherapy regimen employed was cisplatin (CDDP) infused at a dose of 70 mg/m^2^ on days 1 and 29 coupled with continuous 5-fluorouracil (5-FU) infusion at 700 mg/m^2^ per day for 24 h on days 1–4 and 29–32.

### Evaluation of the outcomes, recurrence patterns, and radiation-related adverse events

The patients were consistently evaluated for treatment outcomes and toxicity after radiotherapy. The patients were followed up every 1–3 months in the first post-radiotherapy year and every 3–4 months in the second year. Esophageal endoscopy was performed one month after radiotherapy to assess the efficacy of the treatment, thereby determining a complete clinical response. Physical examination, esophageal endoscopy, measurement of serum concentrations of tumor markers, such as the squamous cell carcinoma antigen, and imaging studies were performed at the follow-up visits. Radiotherapy-related adverse events (AEs) were evaluated using the National Cancer Institute Common Terminology Criteria for AEs version 5.0 [12]. Acute toxicity was monitored weekly during treatment and within three months after radiotherapy. Late toxicity was evaluated subsequently.

### Statistical analysis

Overall survival (OS) was determined as the duration from treatment initiation to death from any cause, and the data of the surviving patients were censored at their last visit. Progression-free survival (PFS) was determined as the duration from treatment initiation to disease progression or death. Survival data were analyzed using the Kaplan–Meier method. Multivariate analyses were performed using a Cox proportional hazards model with forward selection encompassing all baseline factors to address confounding factors, such as age, sex, tumor location, length and invasion, total radiotherapy dose, and concurrent chemotherapy on OS and PFS. All p-values were two-sided, and 95% confidence intervals (CIs) were calculated. Statistical significance was set at p < 0.05. All statistical analyses were performed using the BellCurve for Excel (version 4.04; Social Survey Research Information Co., Ltd).

## Results

### Patients and tumors

Fifty-four patients were included in this study. Table 1 presents the characteristics of all the patients and their tumors. The cohort comprised 49 males and 5 females with a median age of 77 (range: 66–90) years. Based on the Eastern Cooperative Oncology Group scale, all patients had performance status scores of 0–1. The tumor locations were as follows: Ut esophagus, 2 patients; Mt esophagus, 40 patients; and Lt esophagus, 12 patients. The median tumor length was 4.5 (range: 1–16) cm. Tumor invasion was categorized as T1a and T1b in 16 and 38 patients, respectively. The reasons for opting for radiotherapy were significant comorbidities, refusal to undergo surgery, advanced age, and multiple, widespread lesions in 20, 14, 12, and 8 patients, respectively. Twenty-five patients had a history of other carcinomas (46%).

**Table 1:** Characteristics of the patients and tumors

Factor	Number	%
Age (year)		
Range/median	66 − 90/77	
Gender		
Male/female	49/5	90/10
Performance status		
0/1	24/30	45/55
Tumor location		
Ut/Mt/Lt	2/40/12	4/74/22
Tumor length (cm)		
Range/median	1 − 16/4.5	
Tumor invasion		
T1a/T1b	16/38	30/70
Reason for radiotherapy		
Severe comorbidities	20	37
Refusal for surgery	14	26
Old age	12	22
Broad-multiple lesions	8	15
Severe comorbidities	30	56
Cardiovascular disease	15	28
Pulmonary disease	3	6
Diabetes mellitus	10	19
Liver cirrhosis	2	4
History of other carcinoma	25	46
Gastric carcinoma	14	26
Laryngeal carcinoma	2	4
Colorectal carcinoma	2	4
Oral cavity carcinoma	1	2
Parotid carcinoma	1	2
Hypopharyngeal carcinoma	1	2
Bile duct carcinoma	1	2
Renal cell cancer	1	2
Bladder cancer	1	2
Malignant melanoma	1	2

*y.o*. years old, *Ut* upper thoracic, *Mt* middle thoracic, *Lt* lower thoracic

### Treatment

All patients completed the planned IFRT, yielding a 100% completion rate. The fractional dose was set to 1.8 Gy for chemoradiotherapy (CRT) and to 2.0 Gy for patients who underwent IFRT alone. The total doses administered were 50.4 Gy, 59.4–61.2 Gy, and 66–70 Gy in 6, 40, and 8 patients, respectively. To determine the total dose, a 50.4 Gy dose was employed for patients who had completed concurrent chemotherapy, while doses of ≥ 59.4 Gy were administered to patients who had received radiotherapy alone or had not completed concurrent chemotherapy. Eight patients were administered a dose of 66–70 Gy; owing to a relatively small radiation field, the prescribed dose had to be increased. The attending physician made the final decision. The median duration of treatment was 44 (range: 37–53) days. CRT was administered to 33 patients, whereas 21 patients underwent IFRT alone. Among the 33 patients who received chemotherapy, 3, 1, 1, and 28 received nedaplatin (CDGP) + 5-FU, CDDP + Tegafur/Gimeracil/Oteracil (S-1), docetaxel (DTX), and CDDP + 5-FU, respectively.

### Outcomes and recurrence patterns

**Fig. 2:**
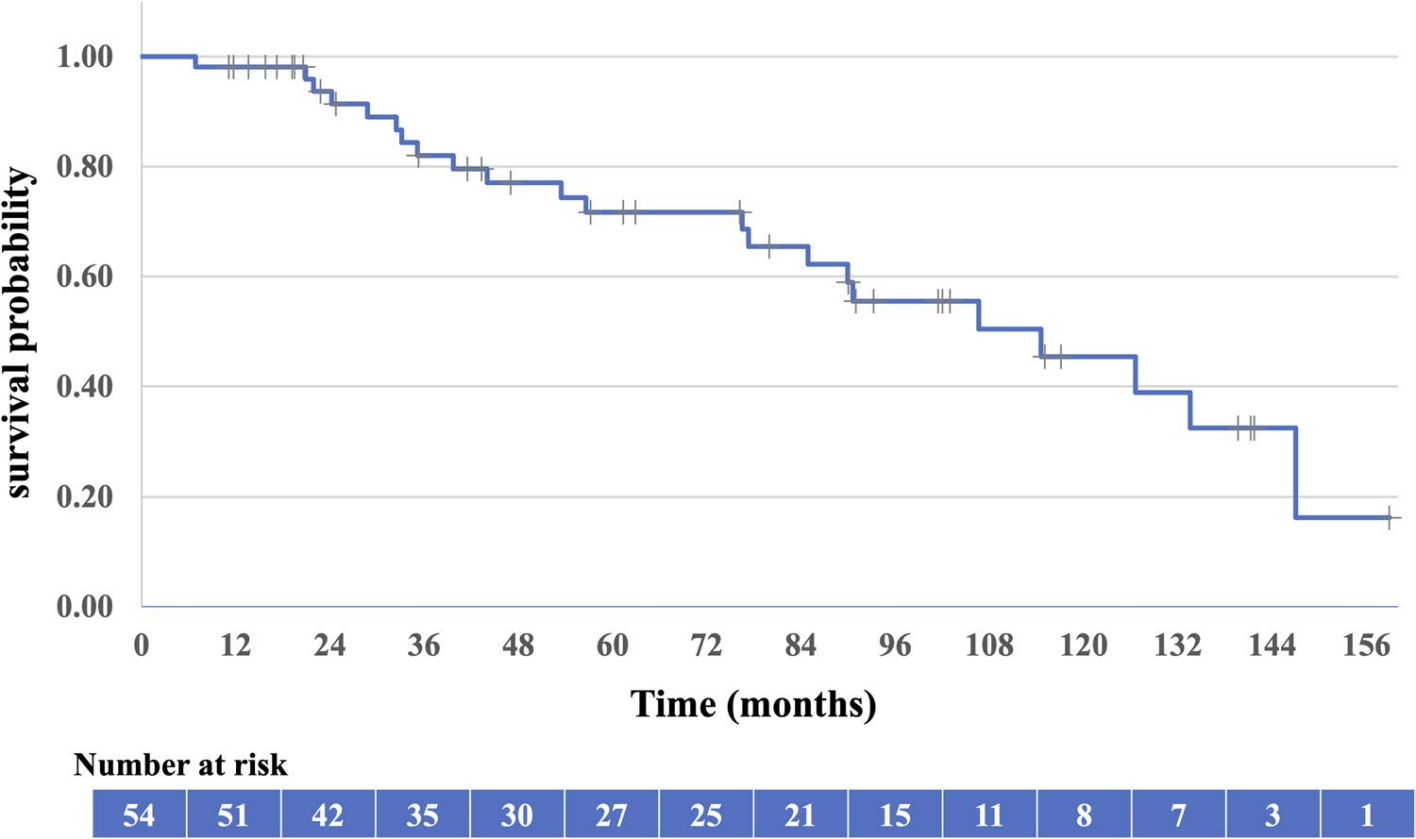
Overall survival of all patients

**Fig. 3:**
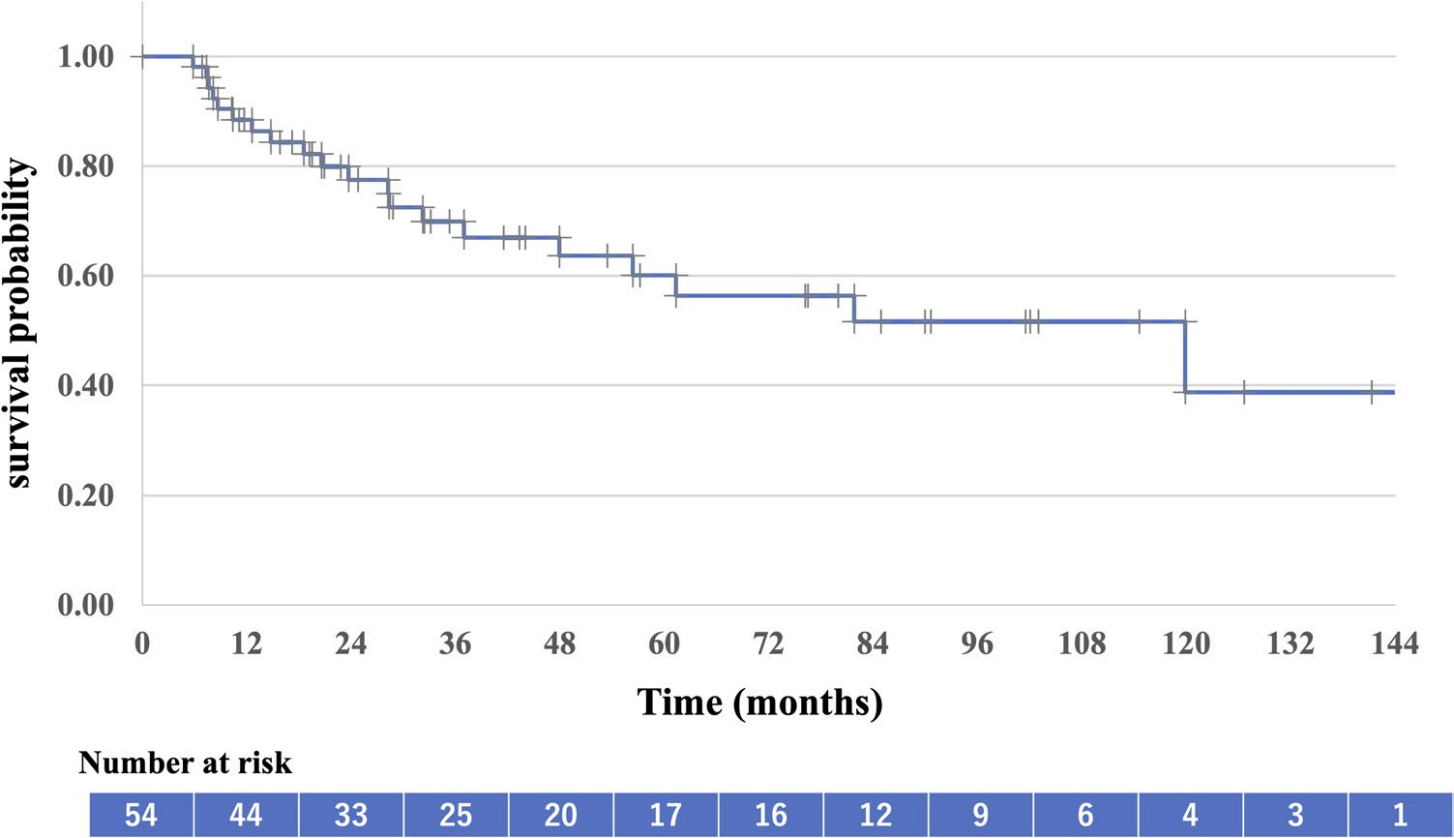
Progression-free survival of all patients

**Fig. 4:**
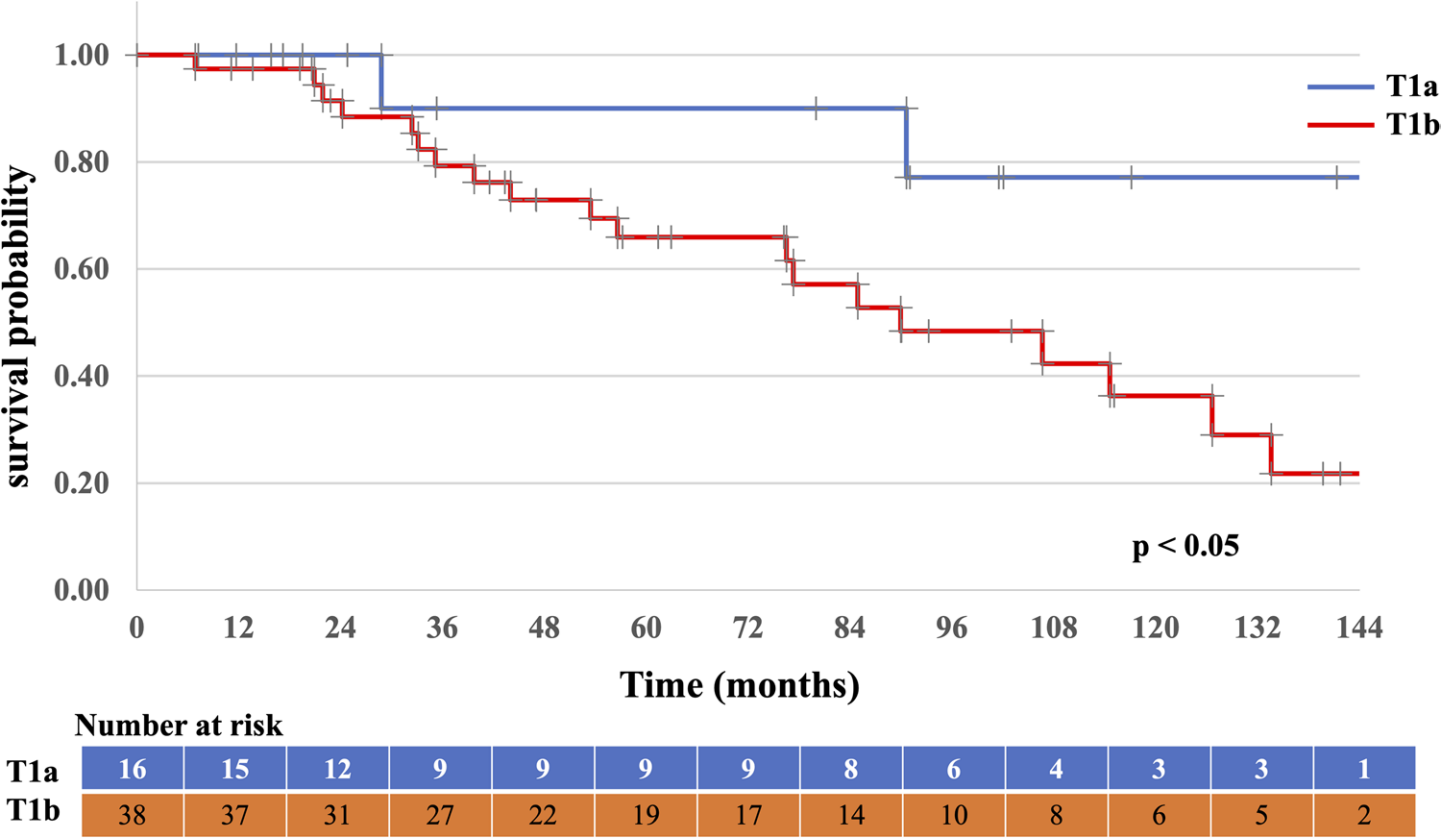
Overall survival of T1a vs. T1b tumors

**Fig. 5:**
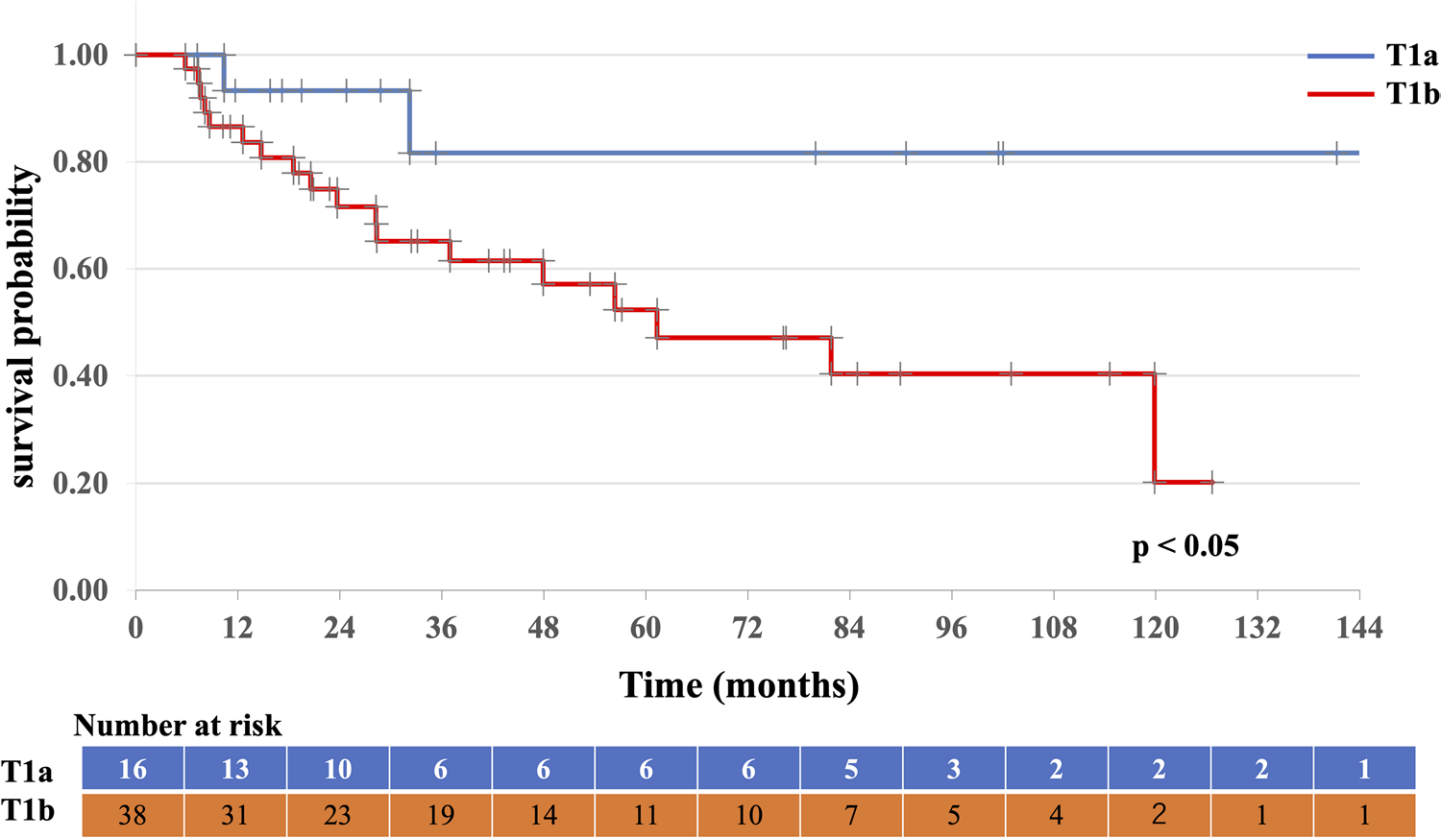
Progression-free survival of T1a vs. T1b tumors

Table 2 presents the outcomes and recurrence patterns. The median follow-up duration was 57 (range: 7–159) months. The median OS was 115 (95% CI 72–157) months, with a 5-year OS rate of 71.7% (95% CI 58.0–85.4%; Fig. 2). The 5-year PFS rate was 60.1% (95% CI 44.6–75.6%; Fig. 3). Patients with T1a tumors had a median OS of 147 (95% CI 102–153) months, a 5-year OS rate of 90.0% (95% CI 47.3–98.5%), and a 5-year PFS rate of 82% (95% CI 58–100%). Patients with T1b tumors had a median OS of 90 (95% CI 75–111) months, a 5-year OS rate of 65.9% (95% CI: 46.8–79.6%), a median PFS of 61 (95% CI 53–88) months, and a 5-year PFS rate of 52% (95% CI 34–71%). The OS and PFS were significantly different between the two groups (Figs. 4 and 5, respectively). Multivariate analysis was used to analyze the prognostic factors associated with OS and PFS; only tumor invasion showed significant differences (Table 3).

**Table 2:** Outcomes and recurrence patterns

	T1 (n = 54)		T1a (n = 16)		T1b (n = 38)		Total (%)
	CR	Non-CR	CR	Non-CR	CR	Non-CR	
Patients	49	5	15	1	34	4	54 (100)
Survival outcome							
Alive/died	29/20	3/2	12/3	1/0	17/17	2/2	32 (59)/22 (41)
Alive without disease	19	2	11	0	8	2	21 (39)
Alive with disease	10	1	1	1	11	0	11 (20)
Died of esophageal cancer	6	2	0	0	6	2	8 (15)
Died of other causes	14	0	3	0	11	0	14 (26)
Site of recurrence							
Local (in-field)	10	3	1	1	9	2	13 (23)
Local (out of field)	2	0	0	0	2	0	2 (4)
Nodal	4	0	0	0	4	0	4 (8)
Distant	1 (liver)	0	0	0	1	0	1 (2)
No failure	32	2	14	0	18	2	34 (63)

*CR* complete response

**Table 3:** Multivariate analysis of prognostic factors associated with OS and PFS

Factor	Overall survival			Progression-free survival		
	P-value	Odds ratio	95% CI	P-value	Odds ratio	95% CI
Age (year), − 75 vs. 76 −	0.45	1.71	0.42 − 7.03	0.97	1.30	0.23 − 4.63
Gender, male vs. female	0.21	0.21	0.02 − 2.45	0.29	0.25	0.02 − 3.24
Tumor location, Ut-Mt vs. Lt	0.95	1.05	0.22 − 5.08	0.07	0.20	0.03 − 1.18
Tumor length (cm), − 4.5 vs. 4.6 −	0.32	2.04	0.50 − 8.29	0.27	0.46	0.12 − 1.84
Tumor invasion, T1a vs. T1b	**0.01**	7.85	1.54 − 39.9	**0.01**	10.7	1.72 − 66.5
Total dose (Gy), 50.4 vs. 59.4 − 70	0.29	2.05	0.55 − 7.71	0.52	0.63	0.15 − 2.59
Chemotherapy, performed vs. not	0.87	0.89	0.22 − 3.89	0.47	0.56	0.12 − 2.67

Bold values are defined as a statistically significant difference

*y.o*. years old, *Ut* upper thoracic, *Mt* middle thoracic, *Lt* lower thoracic

**Fig. 6:**
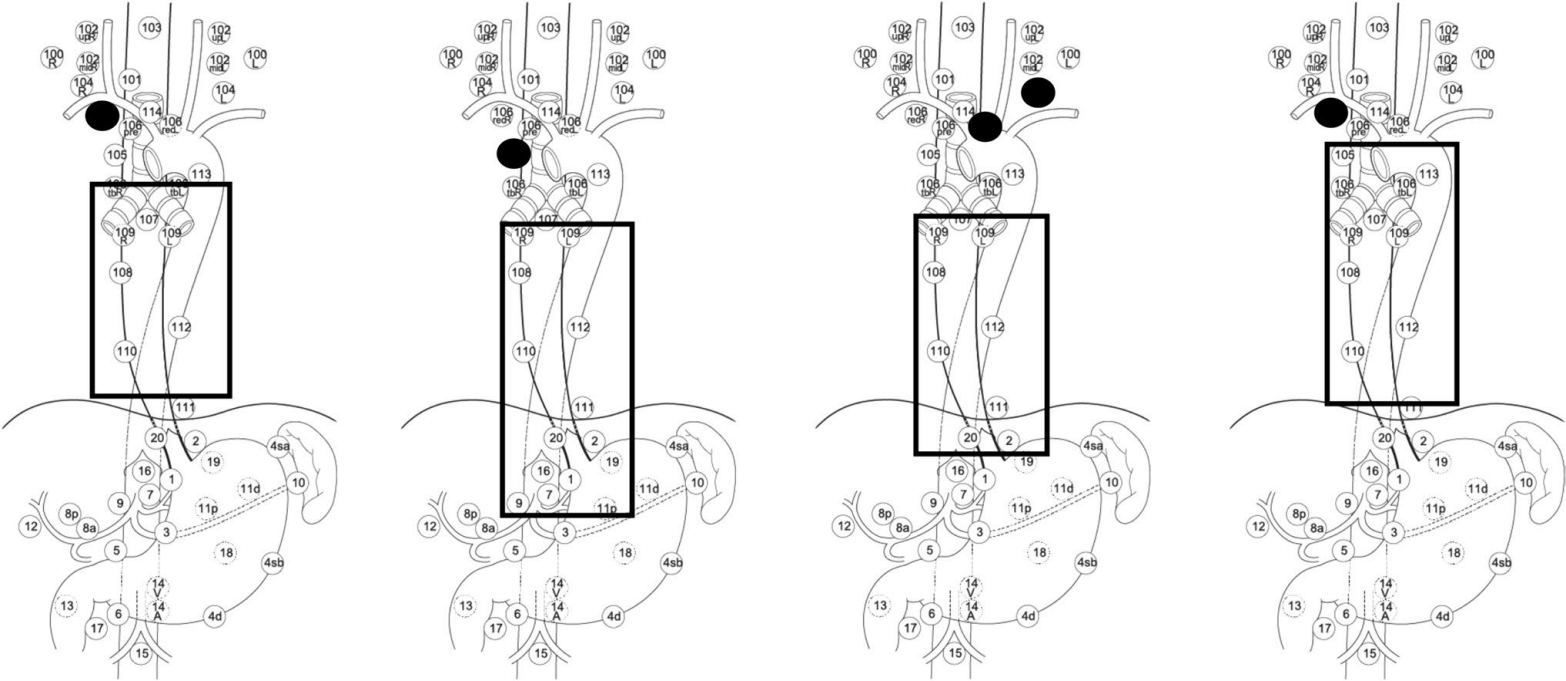
All four out-of-field lymph node recurrences. *The following figures are cited and modified. https://ad123d39pt.smartrelease.jp/wp-content/uploads/2019/12/P131.png

Forty-nine patients achieved a complete response one month after IFRT completion, corresponding to a complete response rate of 90.7%. Recurrence was observed in 20 patients; 13 had in-field recurrence (12 intra-esophageal and one para-esophageal lymph node) and 7 had out-of-field recurrence (4 lymph nodes, 2 esophageal, and 1 liver metastases). All four cases of out-of-field lymph node recurrence were attributed to regional nodes, and all were located headward of the radiation field (Fig. 6).

### Radiation-related AEs

Among acute radiation-related AEs, grade 1 and 2 esophagitis were identified in 28 (52%) and 8 patients (15%), respectively; grade 1 and 2 dermatitis were identified in 7 and 1 patient, respectively; grade 1 gastritis was observed in 3 patients; and grade 1 pneumonitis was observed in 3 patients. For the late AEs, grade 2 esophageal stenosis was observed in 1 patient, grade 1 pneumonitis in 18 patients, grade 1 pleural effusion in 3 patients, and grade 2 pericardial effusion in 4 patients. The incidence of radiation-related AEs was minimal. One patient experienced grade 3 late toxicity, which manifested as pericardial effusion. The patient was a 72-year-old man with Mt-Lt T1a esophageal cancer who underwent 50.4 Gy radiotherapy in 28 fractions with concurrent DTX chemotherapy and was cured. He had a history of gastric cancer surgery and low pulmonary function. The patient developed pericardial effusion ten months after radiotherapy, which affected physiological functions, and pericardiocentesis was performed. The patient was alive and disease-free at 150 months after radiotherapy. Grade 4 or higher toxicities were not observed.

### Salvage treatment

Among the 20 patients who experienced recurrence, 14 received salvage treatment, corresponding to a treatment rate of 70%. The remaining six patients opted for the best supportive care. Among the 13 patients with in-field recurrences, 10 underwent salvage treatment (endoscopic submucosal dissection [ESD], 5 patients; surgical procedures, 2 patients; argon plasma coagulation [APC], 2 patients; photodynamic therapy [PDT], 1 patient). All five patients who underwent ESD achieved a complete response and survived disease-free (OS: 21, 47, 115, 117, and 140 months, respectively). One of the two patients who underwent APC was cured entirely and survived disease-free (OS: 91 months); however, the other developed celiac lymph node recurrence and opted for the best supportive care (OS: 61 months). One of the two patients who underwent surgical procedures recovered completely (OS: 63 months); however, the other had recurrence and died of esophageal carcinoma (OS: 21 months).

Among the four patients with out-of-field lymph node recurrence, two received salvage treatments (radiotherapy, one patient; chemotherapy, one patient); however, both patients presented local esophageal recurrences and died of esophageal carcinoma (OS: 134 and 77 months, respectively).

Two of the three patients with out-of-field recurrences received salvage treatments (ESD, one patient; CRT, one patient). One patient who underwent ESD was cured entirely and survived disease-free (OS: 93 months). However, the other patient, who received CRT, developed local esophageal recurrences and liver metastases, and died of esophageal carcinoma (OS: 159 months).

## Discussion

This study elucidated the long-term outcomes and recurrence patterns of IFRT for esophageal carcinoma, focusing on a localized irradiation field that excludes prophylactic areas. Older patients diagnosed with thoracic ESCC, the predominant type in Japan, were included in this study. Although several clinical trials have investigated the outcomes of IFRT for superficial esophageal carcinoma; most excluded specific demographics, such as age above 65 years or a history of cancer treatment or severe comorbidities. Thus, the outcomes of IFRT in older patients with esophageal cancer previously treated for cancer or those with significant comorbidities remain unclear.

In this study, the 5-year OS rates for stage I were 71.7%, and by T-factor, 90.0% for T1a and 65.9% for T1b, which were favorable outcomes. Only one patient experienced Grade 3 late toxicity (pericardial effusion), and except for this one patient, no Grade 3 or higher AEs were observed. In a multicenter clinical study, the 5-year OS rates of patients with stage I ESCC who received definitive CRT and radiotherapy alone were 67.1% and 44.8%, respectively [6]. In the JCOG9708 phase II trial of CRT for stage I resectable ESCC, the 4-year OS rate was 80.5%, and grade 3/4 late radiation-related AEs were observed in 3.8% of patients (grade 3 ischemic heart disease, one; grade 3 dyspnea, two) [13]. The JCOG0502 trial compared surgery and CRT outcomes for stage I (T1bN0M0) ESCC. Although the randomized portion of the trial was terminated midway due to poor case accrual, the 5-year OS rate for CRT was 85.5% (95% CI 78.9–90.1), and grade 3/4 late radiation-related AEs were observed in 10.5% of patients (grade 4, four patients). Thus, CRT was not inferior to surgery based on the OS of patients with T1bN0M0 ESCC. CRT may be a standard treatment modality for T1bN0M0 ESCC, similar to surgery [14]. Additionally, JCOG0508 reported a 5-year OS rate of 90.9% (95% CI 85.6–94.3%) for patients with esophageal carcinoma who underwent diagnostic EMR, followed by selective CRT for stage I ESCC [15]. Morota et al. reported that grade 3/4 late cardiopulmonary toxicities were observed in 11.5–28% of patients following definitive concurrent CRT for esophageal carcinoma [16]. Previous reports have indicated that acute radiation-related AEs are rarely a clinical issue; nonetheless, late AEs are challenging.

As for the radiation field setting, some studies suggested that IFRT can improve patient prognosis [17, 18], whereas others have favored elective nodal irradiation (ENI) over IFRT [19, 20]. The treatment strategies for older adults represent an important focus of this study, and it is essential to tailor interventions according to each patient's overall health, age, and specific cancer attributes. While observation is sometimes the preferred treatment for this age group, two studies drawing data from the National Cancer Database indicated that patients who were solely observed had a lower OS rate than those who received various treatments [21, 22]. Takahashi et al. examined radiotherapy outcomes in patients aged above 80 years with esophageal carcinoma and reported 3-year OS and PFS of 74.1% and 52%, respectively, for patients with stage I disease [23].

The results of the present study emphasize the importance of judicious decision-making related to the inclusion of prophylactic lymph node regions in the radiation field, especially in facilities inclined to administer CRT or IFRT alone for safety purposes. Although minimal lymph node metastases were observed following IFRT in the present study, an extensive prophylactic irradiation field was not considered imperative. Notably, 46% of our cohort had received cancer treatment previously. Nevertheless, favorable outcomes were observed, partly due to the effectiveness of salvage therapies for recurrent tumors and the low incidence of late AEs, especially pulmonary or cardiac complications. In patients with Mt and Lt lesions, IFRT may not significantly reduce the radiation dose delivered to the heart. However, this study's relatively low incidence of cardiac AEs may be attributable to the four-portal irradiation, which reduced the radiation dose per fraction to the heart, and the low proportion of patients who received concurrent chemotherapy. Interestingly, all four out-of-field lymph node recurrences the irradiated field were observed above the irradiation field. These may have been inadequate pretreatment diagnoses of potential lymph node metastases in the superior mediastinum, including thoracic paratracheal lymph node. Local recurrences and the accuracy of pretreatment diagnosis are closely related. Advanced diagnostic tools, such as PET, have proven helpful in detecting lymph nodes and may be necessary for pretreatment PET, especially in T1b patients. It is also important to predict future lymph node recurrence and set the initial irradiation field to allow salvage irradiation after recurrence in advance. In addition, since local (in-field) recurrences in T1b group are apparently noticeable, it may be necessary to increase the intensity of treatment, if possible, by increasing the prescribed dose or by combining this treatment with chemotherapy.

Post-radiotherapy treatments for residual or recurrent esophageal carcinoma vary, with PDT, EMR, and APC being the primary methods. PDT is minimally invasive, and the widespread use of EMR is limited by technical constraints [24, 25]. In contrast, the efficacy of APC is questionable, although its safety profile is commendable [26].

The treatment modalities for older adults represent an important focus of this study, and it is essential to tailor interventions according to each patient’s overall health, age, and specific cancer attributes. In this study, we propose IFRT for esophageal carcinoma in older patients at a dose of 50.4 Gy in 28 fractions with chemotherapy, while IFRT at a dose of ≥ 59.4 Gy for patients who cannot receive chemotherapy or T1b.

The present study had some limitations. The attending physician had discretion over prescription doses and the decision to use concurrent chemotherapy, as these were not standardized. A comprehensive assessment of late toxicity in esophageal cancer was not possible because the study was retrospective and conducted at a single institution, the patient cohort demonstrated significant differences, and the median follow-up duration was 57 months. Patients with relatively short observation durations were included, and the toxicity analysis may have been inadequate. Such findings should be interpreted with caution. Further studies with a longer follow-up duration, greater sample diversity, and inter-institutional collaborations will strengthen the evidence base. Lastly, while standard radiation approaches, such as an extensive irradiation field, may be optimal for younger patients, they may not align with the needs of older individuals due to the potentially severe AEs.

## Conclusions

The efficacy of IFRT was suggested to be comparable to that of standard treatments, emphasizing its potential as a viable therapeutic modality for superficial ESCC in older adults, especially in those with severe comorbidities. Further studies involving larger sample sizes, longer follow-up durations, and collaboration across institutions will strengthen the evidence in this field, subsequently optimizing clinical decision-making.
